# Facile Atomic‐Level Tuning of Reactive Metal–Support Interactions in the Pt QDs@ HF‐Free MXene Heterostructure for Accelerating pH‐Universal Hydrogen Evolution Reaction

**DOI:** 10.1002/advs.202102207

**Published:** 2021-10-05

**Authors:** Sin‐Yi Pang, Weng‐Fu Io, Jianhua Hao

**Affiliations:** ^1^ Department of Applied Physics The Hong Kong Polytechnic University Hong Kong P. R. China

**Keywords:** 2D materials, electrocatalysts, HF‐free MXene, nanoparticles, reactive metal–support interaction

## Abstract

Supported metallic nanoparticles render highly tunable physical and chemical properties to mixed‐dimensionality materials in electrocatalysts. However, some supports are susceptible to being dissolved in acidic solution or are unstable in ambient air. The development of high‐performance catalysts has been facing the major hurdles of the sluggish activity in alkaline solution and requesting high energy to stabilize the nanoparticles on their supports, challenging the pH‐universality and the applicability of the supported metallic nanoparticles. Here, a one‐step strategy is proposed to modulate the growth of Pt quantum dots (QDs) on HF‐free MXene under atomic‐level by a low‐temperature metal–support interaction reaction. By controllable tailoring in the morphology and strain induced by terminations, Pt (111) QDs with a sub‐nanoscale size of 1.15 nm are grown as 0D/1D heterostructure to overcome the restrictions of employing reduction gas and high annealing temperature. The catalyst exhibits a low overpotential of 33.3 mV for acidic solution, while 65.1 mV for alkaline solution at a specific current density of 10 mA cm^−2^. This study not only paves a scalable pathway to developing cost‐efficient catalysts in moderate conditions, but also demonstrates an effective surface modulation strategy for 0D/1D heterostructures.

## Graphical abstract

Taking advantage of surface‐functionalized MXene and large surface area, a Pt QDs@MXene nanowire is fabricated by a facile method under moderate conditions and shortened synthesis time. After the two‐step transition process, a novel Pt@MXene (0D@1D) nanoheterostructure is formed and renders accelerated kinetics and exhibits a high hydrogen evolution reaction.

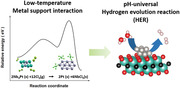

## 1 Introduction

Aroused from the increasing concern to global warming, searching for a reliable energy source has emerged as the most pressing global problem. Hydrogen is regarded as a viable substitute for fossil fuels and is easily produced via an electrocatalytic hydrogen evolution process (HER). Platinum has traditionally been regarded as the most effective electrocatalyst for hydrogen electrolysis in an acidic solution. ^[^ ^1^ ^]^ Nonetheless, the high scarcity and production cost of Pt severely limit its diverse employment in catalysis. ^[^ ^2^ ^]^ Maintaining a high catalytic activity with a minimal amount of precious metal is critical to meeting the rising requirement in the scalable hydrogen industry.^[^ ^3^ ^]^ However, Pt nanoparticles tend to agglomerate together during synthesis, leading to a loss in electrocatalytic activity. Because of their adjustable components, structural and electrical properties, and aggregation‐resistant capabilities, supported metallic nanoparticles enlighten the route to solving the nanoparticles aggregation issue and constitute an exceptional class of materials to electrocatalyst. Taking advantage of its large surface area and unique physical features, 2D materials have garnered a lot of attention in device and energy conversion applications as a promising candidate in catalyst supports.^[^ ^4^, ^5^, ^6^, ^7^ ^]^ However, due to their intrinsic chemical characteristics, 2D‐material‐based metallic nanoparticles always fall into the compromise between pH universality, electrochemical activity, and stability.^[^ ^8^ ^]^ On the other hand, some 2D materials, such as transition metal dichalcogenides, lack the surface‐functionalized as a linker to graft precious metal ions^[^ ^6^ ^]^ or are dissolvable in an acidic environment, such as layered double hydroxides.^[^ ^9^ ^]^

MXene, an emerging class of 2D materials, has engaged an extensive interest in the applications in electrochemistry, biochemistry, and optoelectronics. ^[^ ^10^ ^]^ MXene is generally described by a chemical formula of M*_n_*_+1_X*_n_* T*_x_* (*n* = 1, 2, and 3), and M is an early transition metal from group 13 to 16, X is a carbon, nitride, or carbonitride, whereas T*_x_* is the termination (—F, —Cl, —O, —OH etc.) determined by the etching method. MXenes are commonly synthesized by selective chemical etching of A elements from multilayer MAX materials (A refers group III material, such as Al and Ga) using hydrofluoric acid (HF) or a safer method including NH_4_HF_2_, a mixture of LiF/HCl^[^ ^11^, ^12^ ^]^ and Li/H_2_SO_4_.^[^ ^13^ ^]^ The tunable surface termination influences the physical/chemical properties of MXene and subsequently affects its hydrogen absorption capability in HER.^[^ ^2^, ^14^ ^]^ Nevertheless, typical strategy for synthesizing MXene required high temperature or toxic HF reagent, especially for V‐ or Nb‐based MXene. Furthermore, concentrated HF reagent is a dangerous toxin that can enter the skin and induce systemic poisoning in the human body or death. Additionally, the hydrogen ions which adsorbed on the surface F coverages are not stable, thus may lead to the production of very unstable HF species that desorb from the surface rather than the evolution of H_2_. ^[^ ^14^ ^]^ Recently proposed HF‐free MXene synthesized hydrothermally^[^ ^15^ ^]^ and electrochemically^[^ ^16^ ^]^ with stably terminated —O functional group and the lack of —F termination can prevent competitive behavior between hydrogen and HF species during HER. However, research into the combination of HF‐free MXene with metals is limited. HF‐etched MXene has shown good integrability to mixed‐dimensional materials as well as epitaxial metal growth capabilities. HF‐etched MXene‐support catalyst preparation methods include atomic layer deposition,^[^ ^17^ ^]^ electrochemically immobilized,^[^ ^18^ ^]^ and constructing Pt‐Nb alloy in H_2_ gas reduction by reactive metal–support interaction (RMSI),^[^ ^2^ ^]^ etc. RMSI is a chemical process that takes place at metal–support interfaces and results in the formation of metallic composites, with a high reliance on the reducibility of the supporting material. One effective approach for decreasing the synthesis barrier of alloying Pt to other materials is to reduce metal particle size to single atoms.^[^ ^19^ ^]^ Nonetheless, with the presence of reduction gas (H_2_), high temperatures (>550 °C) are invariably and frequently necessary for these procedures and RMSI. Furthermore, the usage of Pt‐based catalyst is limited by the sluggish kinetics of Pt in alkaline solution.^[^ ^20^ ^]^ Such constraints severely limit the use of MXene‐based catalysts in HER and their potential for scalable production.

With its high durability and surface area, 1D MXene nanowires provide a potential approach for improved electrocatalysts, demonstrating more electrochemical activity than typical 2D MXene nanowires.^[^ ^21^ ^]^ 1D Nb_2_C MXene exhibited high chemical stability and integrability toward metal ions as battery and catalyst.^[^ ^16^ ^]^ Herein, we report a novel Pt QDs@ 1D HF‐free MXene heterostructure fabricated by a low‐temperature RMSI of 150 °C in a vacuum. The morphology and size of the Pt QDs in the low‐temperature RMSI reaction can be controlled using an effective tuning strategy on the morphology and surface termination of the MXene. Under coupling between the porous carbon fiber and the self‐assembly Nb_2_CT*_x_* nanowire, the pH‐universal catalyst of Pt QDs@3D MXene demonstrates a high catalytic activity of 33.3 and 65.1 mV at a specific current density of 10 mA cm^−2^ in acidic and alkaline solution, respectively. This work not only paves an accessible way to RMSI at a lower temperature without the presence of reduction gas but also demonstrates a facile strategy for growing metal QDs on MXene with a pH universal HER activity.

## 2 Results

### 2.1 Characterization and Fabrication of HF‐Free Nb_2_CT*_x_*

The high‐quality HF‐free Nb_2_CT*_x_* MXene nanowires (NWs) were prepared by facile electrochemical etching (E‐etching) method as proposed previously (details in the Experimental Section).^[^ ^16,16^ ^]^ Nb_2_CT*_x_* nanosheets (NSs) were prepared by identical route except for the hydrolyzation step to investigate the effect of morphology of support on MSI. The transmission electron microscopy (TEM) images (Figure S1a,b, Supporting Information) show that Nb_2_CT*_x_* NS holds a lateral size from 400 nm to 1 µm, while the NW owns a length of 100–400 nm with a width of 50 nm on average. Figure S1c,d (Supporting Information) depict a Raman spectrum and X‐ray diffraction (XRD) pattern for confirming the structure of Nb_2_CT*_x_* MXene. The Raman peak and XRD peak respectively shifted from 408 to 388 cm^−1^ and from 12.5° to 11.5°, revealing the deviation between the synthesized MXene and the original MAX phase, caused by the aluminum layer removal.^[^ ^12^, ^16^ ^]^ The thermogravimetric analyzer and linear sweeping voltammetry (LSV), an electrochemical reaction sensitive to changes in surface functionalization, were used to investigate the effect of low‐temperature vacuum annealing on Nb_2_CT*_x_*,^[^ ^14^ ^]^ as presented in Figure S2 (Supporting Information). At the initial drying process from 40 to 100 °C, a plateau appears in the TG curve associated with the enhanced catalytic activity and notably intensified Nb atom signal in the atomic percentage as presented in Figure S2a (Supporting Information). Generally, confined water in a layered material is sensitive to the drying condition below 100 °C.^[^ ^22^, ^23^ ^]^ The decrease in mass and Cl to Nb ratio indicates a change occurred in the surface terminations and more active site exposures due to the desorption of confined water.^[^ ^2^ ^]^ The evaporated Cl composites will serve as an oxidizing agent for the following low‐temperature RMSI. In stark contrast to the initial weight change rate, the surface termination‐sensitive LSV curve (Figure S2b, Supporting Information) shows a significant reduction in weight at higher temperatures, associated with a small change in the Cl to Nb ratio and unchanged catalytic activity.^[^ ^14^ ^]^ The second region of weight loss during drying is reasonable to attribute to the evaporation of surface water.^[^ ^23^, ^24^ ^]^ The thermally sensitive MXene with great controllability and reducibility may be used as a support and is advantageous to the metal growth process.

### 2.2 The Temporal Effect of MSI on Pt QDs Catalyst on Nb_2_CT*_x_* Supports

**Figure 1:**
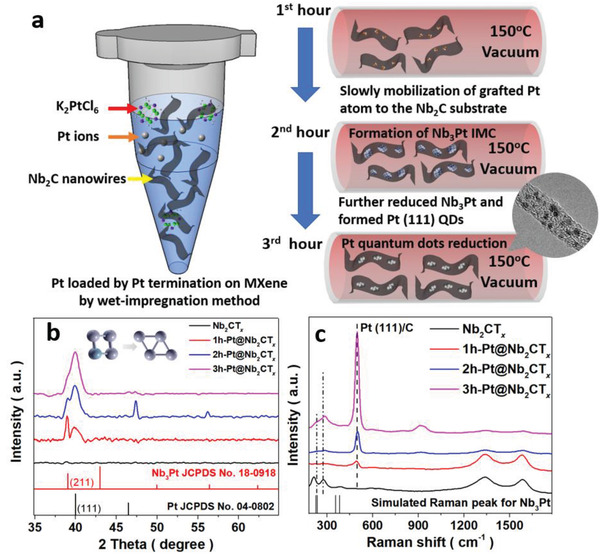
a) Schematics for the fabrication of Pt QDs on Nb_2_CT*_x_* NWs. b,c) Structural investigation for various Pt QDs on MXene by their XRD patterns (b) and Raman spectra (c).

Schematic representation for the synthesis of the Pt@Nb_2_CT*_x_* is sketched in **Figure** **1**a. To synthesize Pt@Nb_2_CT*_x_*, 1 × 10^−3^ m of K_2_PtCl_6_ was combined with 0.5 mg mL^−1^ MXene collides for 30 min with vigorous swirling to induce wet‐impregnation and Pt ion grafting. Pt ions interacted with MXene on the metal–support contact, forming platinum chloride, oxide, and hydroxide from the functional groups‐rich surface of Nb_2_CT*_x_*. The Pt‐grafted Nb_2_CT*_x_* NW/NS was gently heated in vacuum at 150 °C after several washing processes. The XRD and Raman patterns were analyzed to investigate the phase transition of Pt QDs, as illustrated in Figure 1b,c. When compared to the blank Nb_2_CT*_x_* MXene NW, 1h‐Pt@ Nb_2_CT*_x_* exhibits two different peaks at 2*θ* = 39° and 40°. As the duration for vacuum annealing increased, the peak progressively shifted from 39° to 40°, with a substantial decrease in peak ratio from 1.9 to 0.41. According to the JCPDS profiles, the peak at 2*θ* = 39° indicates the presence of Nb_3_Pt (211) nanocrystals, while the peak at 40° indicates the presence of Pt (111) planes. The Raman peaks at 229 and 272 cm^−1^ correspond to the peak shift from Nb—C to Nb/Pt—C bond and the simulated Nb_3_Pt Raman peak, respectively. The formation of Raman peaks at 498 cm^−1^ attributed to the Pt(111)/C vibration^[^ ^25^ ^]^ rather than Pt(100)/C vibration,^[^ ^26^ ^]^ indicating the success of controlled Pt growth on MXene during the annealing process.

**Figure 2:**
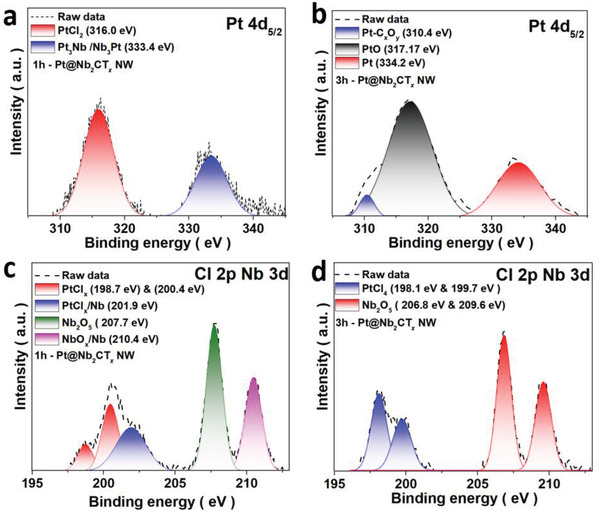
a,b) The XPS patterns of 1h/3h‐Pt@Nb_2_CT*_x_* NW catalysts.The Pt 4d region of the XPS spectrum for 1h‐Pt@Nb_2_CT*_x_* NW (a) and 3h‐Pt@Nb_2_CT*_x_* (b) NWs. c,d) Cl 2p and Nb 3d states XPS spectrum of Pt@ Nb_2_CT*_x_* NW annealed for 1 h (c) and 3 h (d).

The interfacial effect between platinum and MXene was further investigated by ex situ X‐ray photoelectron spectroscopy (XPS) as shown in **Figure** **2**. Figure S3a (Supporting Information) shows the Pt 4f of the XPS spectrum of the 1h‐Pt@Nb_2_CT*_x_* NW catalyst. The strong pair of doublet (72.7 and 75.7 eV) was assigned to Pt (II) chemical state in Pt(OH)_2_ and PtCl*_x_*. In comparison to the XPS pattern for 1h‐Pt@ Nb_2_CT*_x_* NW, the XPS pattern for 3h‐Pt@ Nb_2_CT*_x_* NW illustrated a deconvolution of Pt 4f XPS doublet (4f_7/2_ and 4f_5/2_) and gave rise to two pairs of strong doublets (Figure S3b, Supporting Information). The first set of doublets (71.0 and 74.9 eV) was due to metallic Pt and the second set of doublets was observed at binding energy 1.4 eV higher than Pt(II).^[^ ^1^ ^]^ Similar pair of doublet in 1h‐Pt@Nb_2_CT*_x_* NW catalyst was attributed to the surface Pt(II) species. The Pt species with higher binding energy are most likely caused by Pt—Nb alloying, owing to extra coulombic interaction between the photon‐emitted electron and the ion core (Figure 2a,b). The result supports the reduction of Pt from Pt(II) to Pt_0_. Meanwhile, the binding energy of Cl shifted from 202 to 200 eV (Figure 2c,d), implying a reduction in the oxidation state, which contrasts with the almost no change of the binding energy of Nb. Furthermore, the significant binding energy shifts in all XPS spectra (Figure S4, Supporting Information) are caused by the reduction in oxidation state during vacuum annealing. The Nb/Pt‐Cl species generated in the initial state is consistent with the XRD, Raman, and XPS data. Some of the bimetal species were reduced by the reactive Cl reduction agent, while Nb_3_Pt and Pt species remained after washing the dissolvable Cl species.

**Figure 3:**
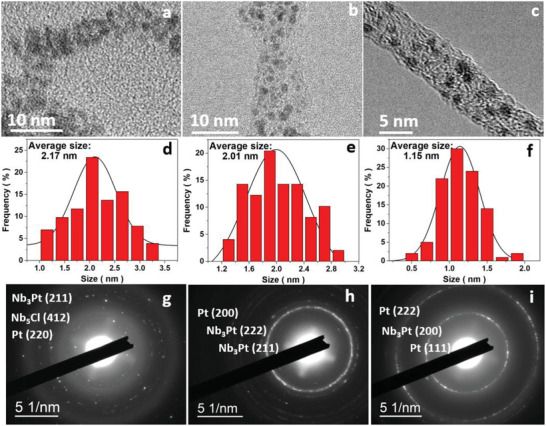
TEM and SAED images for various catalysts synthesized at different conditions. a–c) TEM images for the Pt@Nb_2_CT*_x_* catalysts annealed for 1 h (a), 2 h (b), and 3 h (c). d–f) The distribution of the Pt@Nb_2_CT*_x_* catalysts annealed for 1 h (d), 2 h (e), and 3 h (f). g–i) SAED patterns for Pt@Nb_2_CT*_x_* catalysts annealed for 1 h (g), 2 h (h), and 3 h (i).

The high reducibility of Nb_2_CT*_x_* offers a highly tunable platform to the RMSI reaction and the particle size engineering of the Pt QDs. In addition to the phase transition of the interfacial Pt, the size of Pt QDs is also significantly affected by the annealing time. The particle size of the catalysts was first studied by high‐resolution (HR) TEM (**Figure** **3**) and the samples reached a quantum‐level particle size of 2.17, 2.01, and 1.15 nm for 1, 2, and 3 h annealing, respectively. It is worth noting that the longer annealing period after 3 h may result in substantial nucleation; a 6 h‐annealed sample revealed that metal nanoparticles began to cluster together because of the nucleation activity of the Pt nanoparticles as shown in Figure S5 (Supporting Information). The morphological result holds a strong consistency in which the size of the Pt QDs reduced as the Pt to Nb ratio decreased (Figure S6, Supporting Information). The HRTEM image reveals a different species grown on 1h‐Pt@Nb_2_CT*_x_* (Figure S7, Supporting Information). It corresponds to the transition from Pt/Nb alloy to Pt nanoparticles, while the selected‐area electron diffraction (SAED) pattern (Figure 3g) of 1h‐Pt@Nb_2_CT*_x_* showed the lattice plane of NbCl_5_ (412), Pt (200), and Nb_3_Pt (211) nanocrystals on the 1h‐Pt@Nb_2_CT*_x_* catalyst, demonstrating the coexistence of the Pt/Nb alloy, Pt, and the reactant agents. The SAED pattern of the 2h‐Pt@Nb_2_CT*_x_* showed two sets of (211) and (222) reflection corresponding to Pt/Nb alloy as shown in Figure 3h. After 3 h of low‐temperature annealing as imaged in Figure 3i, a clear SEAD pattern reflected the (111) and (222) plane from the nanoparticles, indicating Pt nanoparticles were grown on MXene with a diminished amount of Nb/Pt alloy. The origin of the two‐step transition may be attributed to the unstable state of the Nb/Pt alloy.^[^ ^27^, ^28^ ^]^ Upon heating, —Cl functionalizes were vaporized in the form of chlorine gas and accelerated the phase separation of the Nb_2_C substrate and Pt QDs possibly caused by the inhomogeneous adsorption of metal pairs. The factor of specific area has a significant influence on the size of the nanoparticles, similar to the temporal effect of annealing. As demonstrated in previous studies, the specific surface area of MXene nanowire is typically larger than that of MXene nanosheet,^[^ ^16^ ^]^ and the specific surface area of the support influences the extent of RMSI, concentration of defects and the growth of metal particles.^[^ ^29^ ^]^ In contrast to the narrow size distribution of Pt QDs on Nb_2_CT*_x_* NW, Pt@Nb_2_CT*_x_* NS demonstrates a broad and nonuniform distribution as shown in the TEM images under identical synthesis condition, with a relatively bulky Pt nanoparticle (≈7.14 nm) on the surface of the Nb_2_CT*_x_* NS (Figure S8, Supporting Information). Typically, zerovalent atoms of platinum group metals are mobile at elevated temperature and would agglomerate into larger particles (>10 nm), thus losing their catalytic efficiency in the traditional synthesis method due to their bulk size.^[^ ^30^ ^]^ The low‐temperature RMSI provides a pathway to synthesize a nanoparticle achieving a quantum dot (1.15 nm) level with fine control.

### 2.3 The Effect of Composition and Crystal Structure on MSI

**Figure 4:**
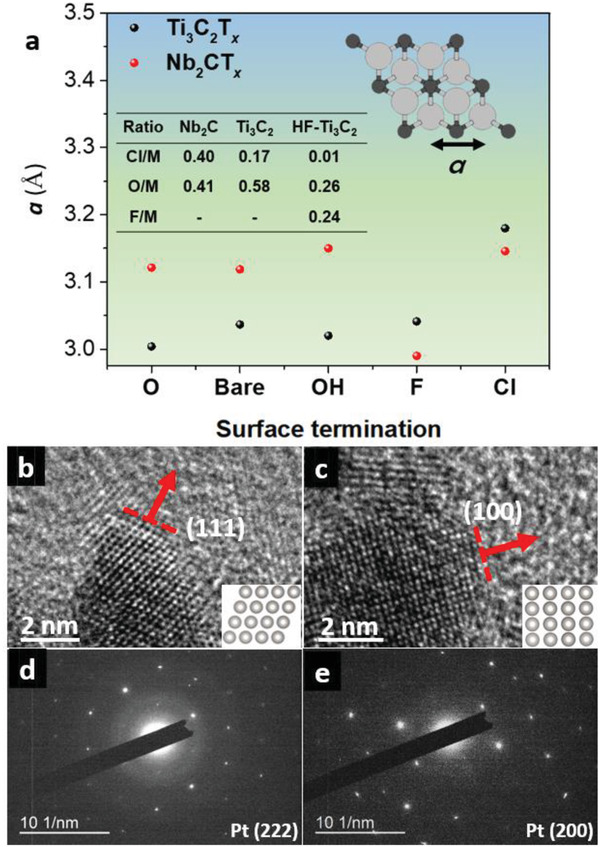
a) The dependence of the in‐plane lattice constant *a* (equivalent to Ti–Ti and Nb–Nb) for Nb_2_CT*_x_* and Ti_3_C_2_T*_x_* MXenes on the chemical nature of the surface group T*_x_*. b,c) HRTEM of Pt growth on E‐etched MXene (b) and HF‐etched MXene (c) and d,e) the corresponding SEAD images for E‐etched MXene (d) and HF‐etched MXene (e).

The crystal structure and composition of the support mutually affect the growing environment for the metal nanoparticles.^[^ ^29^ ^]^ Due to the zero‐energy bandgap, Nb_2_C with all terminations is metallic (as shown in Figure S9, Supporting Information) and holds a similar work function to Pt (Nb_2_C in ≈5.8 eV and Pt in 5.65 eV, Ref. ^[^ ^31^ ^]^). To study the MXene effect using different synthesis route, i.e., HF‐free MXene and HF‐etched MXene (denoted as 3h‐Pt@Ti_3_CT*_x_* and 3h‐Pt@HF‐Ti_3_CT*_x_*, respectively), we calculated the lattice structures employing first‐principles quantum molecular dynamics and a structure optimization procedure using the Quantum ESPRESSO code. According to the structural calculation results, —F‐functionalized MXenes have a smaller lattice parameter than —Cl functionalized MXenes (as shown in **Figure** **4**a), which is due to the high electronegativity and related in‐plane tensile strain. Generally, a misfit in lattice between the nanoparticles and the support generates internal strain and defect. Accordingly, utilizing the misfit can modify the morphology of the nanoparticles. It is worth noting that the HF‐free MXene with —Cl usually holds a larger in‐plane lattice constant for both Nb_2_CT*_x_* and Ti_3_CT*_x_* in the density functional theory (DFT) calculation, coherent with the TEM results (Figure S10, Supporting Information) and literatures.^[^ ^32^, ^33^ ^]^ In Figure 4a,b, an observable difference is demonstrated in the growth of Pt nanoparticles. For the E‐etched 3h‐Pt@Ti_3_CT*_x_*, Pt (111) was successfully grown on the surface. In sharp contrast to the E‐etched MXene, Pt (100) tended to grow on HF‐Ti_3_CT*_x_* catalyst. Figure S10 (Supporting Information) images that the HF‐etched MXene owns a small lattice parameter compared to the HF‐free MXene, consistent with the DFT result. Additionally, the energy‐dispersive X‐ray spectroscopy (EDS) result (Figure 4a) depicts the HF‐etched MXene was mainly functionalized by —F and —O, while the HF‐free MXene were majorly functionalized by —Cl and —O. The result elucidates the reason for Pt tending to grow on the HF‐etched MXene in (100) rather than (111) as the comparable *a*‐lattice parameter on Pt (100) plane and —F functionalized MXene (0.281 and 0.299 nm). In the SAED for Pt growth on HF‐free MXene, the lattice plane estimated a lattice parameter of 0.148 nm corresponds to Pt (222) plane, whereas the SAED for Pt growth on HF‐etched MXene with a lattice spacing of 0.198 nm attributes to Pt (200) plane in Figure 4d,e. Raman spectroscopy was conducted to investigate the origin of the difference between two supports. In Figure S11 (Supporting Information), an intense signal rises from metal oxide at 150 cm^−1^ and assigned to titanium oxide.^[^ ^16^ ^]^ As the previous discussion of the stability and the formation of the oxide film on HF‐etched MXene, it is inevitable to grow oxide film on MXene. The higher oxidizing rate of HF‐etched MXene in ambient air was likely contributed to the active —F termination.^[^ ^14^ ^]^

### 2.4 The Electrochemical Performance of the MSI Controlled MXene

**Figure 5:**
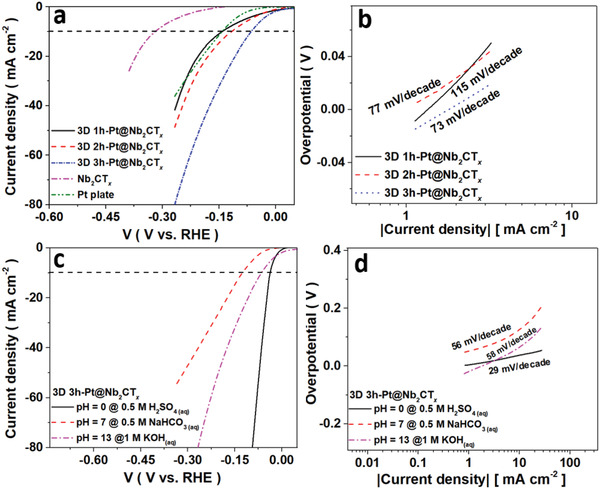
a) LSV for 3D Pt@Nb_2_CT*_x_* with different synthesis time and b) the corresponding Tafel plot in alkaline solution. c) The LSV for 3D 3h‐Pt@Nb_2_CT*_x_* in different electrolytes and d) the corresponding Tafel plot.

Constraining from the low conductivity in metal oxide, the scope and progress on the tuning of MSI through support morphology in electrocatalysis are severely restrained. Inheriting from the intrinsic self‐assembly ability from the Nb_2_CT*_x_* NW to carbon‐fiber cloths (CFCs), Pt@ Nb_2_CT*_x_* was tested on the CFC support as demonstrated in Figure S12 (Supporting Information) to evaluate the catalytic activity of the catalysts. The electrochemical activity of the catalysts greatly enhanced by controllable tuning in the surface termination, morphology, and composites. **Figure** **5** depicts the difference between the 3D Pt@ Nb_2_CT*_x_* catalysts. The 3D 3h‐Pt@Nb_2_CT*_x_* composite exhibits a low overpotential of 33.3 mV for an acidic solution, while 65.1 mV for an alkaline solution at a specific current density of 10 mA cm^−2^, as shown in Figure 5a,b. Accordingly, such HER catalytic ability is comparable to state‐of‐the‐art HER catalysts including Pt plate, commercialized Pt/C^[^ ^34^ ^]^ and the single atom doped MXene catalyst with much lower synthesis temperature and mild fabrication condition.^[^ ^18^ ^]^ The low kinetics of Pt nanoparticles in alkaline solution was accelerated by using pH universal HF‐free Nb_2_CT*_x_* substrate and ranks in the top within the existing catalyst as compared in Table S1 (Supporting Information). The result also reveals that the utilization of 1D HF‐free MXene reduced the synthesis temperature and resulted in a smaller particles size down to QDs level.

The catalytic activity subsequently boosted due to the Tafel‐reaction determining electrochemical behavior on the Pt particles in QD level. The catalytic activity of the MXene in the different electrolyte was depicted in Figure 5c,d and high Tafel slopes were presented in all curves. In the Tafel reaction, the HER activity heavily depends on the double‐layer capacitance, and thus a diminished metal particles size is beneficial to the catalytic activity. The increase of the electrochemically active site is also impactive to the H_2_ coverage on the catalyst.^[^ ^35^ ^]^ Furthermore, increased crystallite and decreased crystal size speed up the surface kinetics, and it is advantageous to the hydrogen absorption and hydrogen evolution reaction. We postulate that the enhanced HER performance of the 3D Pt@ Nb_2_CT*_x_* catalyst is due to the unique chemical synthesis approach's distinct heterojunction MXene–Pt structure and the synergistic influence of MXene–Pt since the HER performance is larger than that of individual Pt or MXene. The HER performance of the catalyst improves as the electrolyte changes from acidic to basic to neutral. Because there is no water association step in acidic solution (as equated in the Supporting Equations), the reaction proceeds immediately through the Volmer–Tafel step, which promotes a fast kinetics. Similarly, because H_2_O dissociation is kinetically favorable on the Nb_2_C substrate in basic solution, it offered a shorter pathway for completing the water dissociation process, and the following reaction was completed by a fast Volmer–Tafel reaction. The reaction mechanism of catalyst in the neutral electrolyte is similar to that in an acidic solution,^[^ ^36^ ^]^ but with a lower concentration of hydrogen ions, whereas the lower conductivity of the solution also limits the high activity of the catalyst and exhibits a lower overpotential of 124 mV at a specific current density of 10 mA cm^−2^ in slower Volmer–Heyrovsky reaction. For comparing the effect of the mismatch for electrochemical activity in the band position and crystal structure between semiconducting supports and the metal NPs, the electrochemical impedance of 3h‐Pt@HF‐Ti_3_C_2_T*_x_*, 3h‐Pt@Ti_3_C_2_T*_x_*, and 3h‐Pt@V_2_CT*_x_* was exanimated in Figure S13a (Supporting Information). Figure S13b (Supporting Information) shows a higher overpotential in the HER curve, and a higher resistance appeared in the electrolyte/electrode interface for 3h‐Pt@V_2_CT*_x_*, demonstrating the merits of Nb_2_CT*_x_* in high conductivity and barrier‐free fast kinetics. Furthermore, 3h‐Pt@Ti_3_C_2_T*_x_* exhibits transmission line behavior, indicating sluggish ion diffusion transfer activity. MXene that has been E‐etched has a low diffusion resistance and a high ion transfer activity. It is noted that the contact resistance is nearly identical for Pt@MXene catalysts due to the dominated conductivity of Pt nanoparticles. The difference in LSV is mainly attributed to the phase difference of Pt nanoparticles, where Pt nanoparticles growth with (111) are facilitative to redox‐active electrochemical reaction in catalysis than (100) direction where slower kinetics generally observed in Pt composites, especially for the metal phase Pt (100).^[^ ^37^ ^]^ The results indicate that changing the surface functional group and the MXene composition can affect the surface morphology and the composite of grown nanoparticles. Thus, it is impactive to the electron transfer kinetics and electrochemical activity. The 3h‐Pt@Nb_2_CT*_x_* catalyst exhibits high kinetics in a universal pH electrolyte by taking advantage of the charge‐transfer kinetics‐favored architecture.

## 3 Conclusion

Without the use of reduction gases or high temperatures, HF‐free MXene provides a highly tunable platform for MSI reactions. The MSI tuning effect on the MXene has been demonstrated structurally, morphologically, and spectroscopically. The misfit in band position and crystal structure corresponds to a different structure in the grown nanoparticles. The surface functional group has a significant influence on the lattice structure and affects the MSI on the HF‐etched and HF‐free MXene based on DFT calculations and structural analysis. The electrochemical activity of the heterostructure Pt@MXene catalyst increases as the phase transition from Pt/Nb alloy to Pt nanoparticles occurs. With the assistance of the hydro‐absorption‐friendly HF‐free MXene, the 3h‐Pt@Nb_2_CT*_x_* demonstrates strong kinetics toward HER in both alkaline and acidic solutions, beyond the state‐of‐art and commercialized Pt‐based catalysts. Furthermore, the strong covalent interaction between the metal QDs and the support provides 3h‐Pt@Nb_2_CT*_x_* with great robustness and stability toward HER. This research demonstrates the ease in which RMSI can be tuned on 2D MXene support and opens the door to a novel scalable and safe technique for developing a heterostructure catalyst without the usage of explosive reduction gas.

## 4 Experimental Section

### Materials and Reagents

Niobium, titanium, and vanadium aluminum carbide (200 mesh, 99% purity) were purchased from Laizhou Kai Ceramic material Co., Ltd. Hydrochloride, potassium hydroxide, lithium fluoride, sulfuric acid, and sodium hydroxide were purchased from Sigma. Polyvinyl alcohol (88%) was purchased from Aladdin‐reagent Co., Ltd. All materials were used as received without further purification.

### Purification of Carbon Fiber Cloths

The CFCs were purified as previously reported.^[^ ^16^, ^38^ ^]^ The CFCs were cleaned with ethanol and acetone before being submerged in the HNO_3_ solution for 6 h under reflux. The NaOH solution neutralized the CFCs, which were then washed multiple times with water and dried in a 60 °C oven.

### Synthesis and Collection of the 1D/2D MXene

The hydrolyzed Nb_2_AlC was first fabricated as a precursor for the MXene NW. In the typical route, 1 mL of 6 m KOH solution was added to 100 mg Nb2AlC, followed by 4 h of intense stirring at 50 °C. MXenes were produced without the need of HF^[^ ^16^ ^]^ via 3D electrode preparation. The as‐diminished MAX phase was mixed with carbon black in a 95:5 ratio and uniformly drop‐cast onto the CFC substrate. The Al layer was selectively removed by anodizing the MAX phase precursor for 4 h at 1 V in 0.5 m HCl electrolyte. The process was identical for the 2D MXene, but no hydrolyzation step was involved for the precursor. After 4 h, the as‐synthesized MXenes were placed in a test tube. Pipette was used to collect 80% of the supernatant, and the remnants were discarded. The 1D MXene was purified by collecting the supernatant obtained after centrifugation at 3000 rpm for 5 min.

### Preparation of Pt QDs@ Nb_2_CT*_x_* NW

All catalysts were fabricated by a similar approach, as reported previously. MXenes (1 mg) were well dispersed in 2 mL of NaOH solution (1 m) and intensely stirred by magnetic bars for 1 h. The 0.1 × 10^−3^ m of K_2_PtCl_6_ prepared as the Pt seed was mixed with the NaOH–MXene followed by several times of wash by the deionized (DI) water.

### Drop‐Casting of Nb_2_CT*_x_* NW/Pt Metal Dispersed Nb_2_CT*_x_* NW/NS on 3D CFC Backbone

The as‐synthesized MXene colloids were dispersed in a 0.5 mL mixture of water and alcohol (in a ratio of 1:1). The CFCs were heated at 50 °C in air. Afterward, 0.2 mL of MXene colloids was transferred by pipette and then uniformly drop‐cast on the porous CFC. Upon gentle heating, the nanowire networks were formed gradually by layer to layer through several times drop‐casting procedure.

### DFT Calculation for Bandgap and Lattice Structure

All first‐principles simulations based on DFT were carried out using the Quantum Espresso software package. The exchange and correlation energies were calculated using a local‐density approximation as parameterized by (DFT‐PBE). The plane wave's kinetic energy cutoff is 700 eV and a vacuum layer of 15 Å is used. For cell optimization and relaxation, only the *x* and *y* dimensions change iteratively (i.e., 2D *xy* constraint) for MXene. The dipole and vdW interactions were considered. For MXenes, a 12 × 12 × 1 Monkhorst k‐point grid was used, while for Nb_3_Pt, a 12 × 12 × 12 Monkhorst k‐point grid was used. The vibrational frequencies for light scattering were determined using dynamic matrices in a finite‐difference method.

### Statistical Analysis of Size Distribution of Nanoparticles

For data preprocessing, no transformation or normalization was employed. The data are displayed in a bar chart, and the sample size for each statistical analysis is 50. Gatan Digital analytic software was used to analyze the TEM images to determine particle size.

## Conflict of Interest

The authors declare no conflict of interest.

## Supporting information

Supporting Information

## Data Availability

Research data are not shared.
