# Supplementary material for: Facile Atomic‐Level Tuning of Reactive Metal–Support Interactions in the Pt QDs@ HF‐Free MXene Heterostructure for Accelerating pH‐Universal Hydrogen Evolution Reaction
Source: Adv Sci (Weinh). 2021 Oct 5;8(22):2102207. doi: 10.1002/advs.202102207 (PMC8596115; doi:10.1002/advs.202102207)
Supplement: Supplementary file 1 — Supporting Information [file ADVS-8-2102207-s001.pdf]

© 2021 Wiley-VCH GmbH

ADVANCED SCIENCE
Open Access

Supporting Information

for *Adv. Sci.*, DOI: 10.1002/advs.202102207

Facile Atomic-Level Tuning of Reactive Metal–Support Interactions in the Pt QDs@ HF-Free MXene Heterostructure for Accelerating pH-Universal Hydrogen Evolution Reaction

*Sin-Yi Pang, Weng-Fu Io and Jianhua Hao**

Supporting Information

Facile Atomic-Level Tuning of Reactive Metal–Support Interactions in the Pt QDs@ HF-Free MXene Heterostructure for Accelerating pH-Universal Hydrogen Evolution Reaction

*Sin-Yi Pang[1], Weng-Fu Io[1] and Jianhua Hao[1]**

[1]Department of Applied Physics, The Hong Kong Polytechnic University, Hong Kong, P. R. China

*Corresponding author. Email: jh.hao@polyu.edu.hk

Material characterization

Morphology of the Nb_2CT_x NW sample was imaged using TEM (JEOL 2100F Transmission Electron Microscope, Japan) with 200 kV accelerating voltage. Elemental characterizations and the morphologies of the catalysts were investigated by scanning electron microscope (SEM, JEOL Model JSM-6490) with an energy-dispersive X-ray (EDX) spectrometry system. Raman spectra of the samples were taken from Witec Confocal Raman system at 532 nm wavelength diode laser. X-ray photoelectron spectroscopy (XPS) was examined by Thermo Scientific™ Nexsa™ X-Ray photoelectron spectrometer system, equipped with monochromatic and microfocus Al K_α X-ray source (1486.2 eV). All samples were freshly prepared and washed before being measured in the XPS section. Powder X-ray diffraction

(XRD) patterns of MXene were measured using a Rigaku smart lab 9 kW (Rigaku, Japan) coupled to a 2D detector with Cu K_α radiation (λ = 0.154 nm).

HER and EIS measurement

The electrochemical characterizations were performed in 1 M KOH (aq), 1 M H_2SO_4 (aq), and NaCl (aq) electrolytes utilizing a Solartron Electrochemical workstation with a standard three electrode setup. A glassy carbon electrode was used as the working electrode, while a carbon rod and a standard calmer electrode (SCE) filled with saturated KCl solution served as the counter and reference electrodes, respectively. The catalysts were dispersed in 0.5 mL D.I. water and drop-casted onto a GC electrode without any further additives, with a mass loading of 0.1 mg cm^{-2} on the working electrode for all catalysts with a thickness of 80 µm whereas Pt plate in a thickness of 100 µm. The HER LSVs were evaluated at a scan rate of 5 mV s^{-1} with no external gas purging, and the EIS curve was scanned from 1 MHz to 10 mHz with a voltage amplitude of 4 mV *vs.* SCE.

Supporting equations

In alkaline medium, HER involves three steps:(i) the Volmer step, (ii) water dissociation, and (iii) creation of a reactive hydrogen intermediates. And the processes were equated as following[1]:

Volmer reaction – Discharge reaction (120 mV dec^{-1}):

$$H_2O + e^- \rightarrow M\text{-}H + OH^-$$

Tafel reaction – Combination reaction (30 mV dec^{-1}):

$$M\text{-}H + M\text{-}H \rightarrow H_2 + 2M$$

Heyrovsky reaction – Ion + atom reaction (40 mV dec^{-1}):

$$H_2O + e^- + M\text{–}H \rightarrow H_2 + OH^- + M$$

In an acidic electrolyte, the proton/hydrogen reduction process can take place via the Volmer-Tafel or Volmer-Heyrovsky pathways. The following is the mechanistic route of HERs in acidic solution:

Volmer adsorption step:

$$M + H^+ + e^- \rightarrow M\text{-}H$$

Tafel dissociation desorption step:

$$2M\text{-}H \rightarrow H_2 + M$$

Heyrovsky desorption step:

$$M\text{-}H + H^+ + e^- \rightarrow H_2 + M$$

*Where M represent the catalyst.

Supporting tables

Table S1. Comparison of the catalytic performance of different MXene catalysts

Catalysts	Overpotential @ 10 mA/cm^2 (mV)	Tafel slope (mV/dec)	Type of electrolyte	Metal particle size (nm)	Ref.
3D 3h-Pt@Nb_2CT_x	33.3	29	Acidic	1.15	This work
	61.5	58	Alkanline	1.15	This work
3D-Nb_2CT_x NW	400	117	Alkanline	-	[2]
Pt/$Ti_3C_2T_x$-550	32.7	32.3	Acidic	6.6	[3]
TBA-$Ti_3C_2T_x$-Pt-20	55	65	Acidic	2	[4]
$Mo_2TiC_2T_x$–Pt_{SA}	30	30	Acidic	-	[5]
Pt NWs/SL-$Ni(OH)_2$	>75	-	Alkaline	1.8	[6]

Supporting Figures

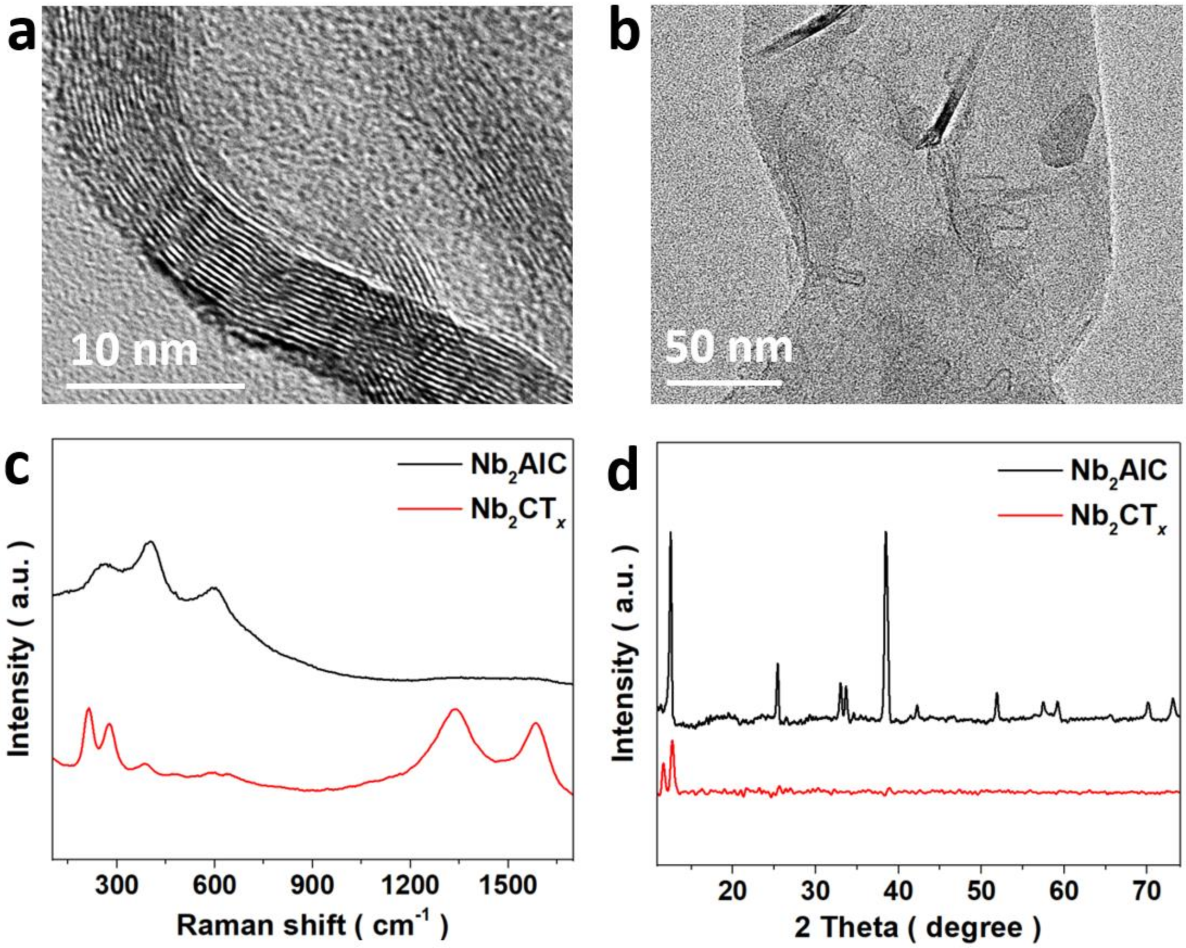

Figure S1. Structural and morphological characterization of pristine MXene Nb_2CT_x supports. TEM images for (a) Nb_2CT_x NW and (b) Nb_2CT_x NS. (c) Raman spectroscopy and (d) X-ray diffraction pattern confirms the selective etching of the MXene from the MAX phase materials.

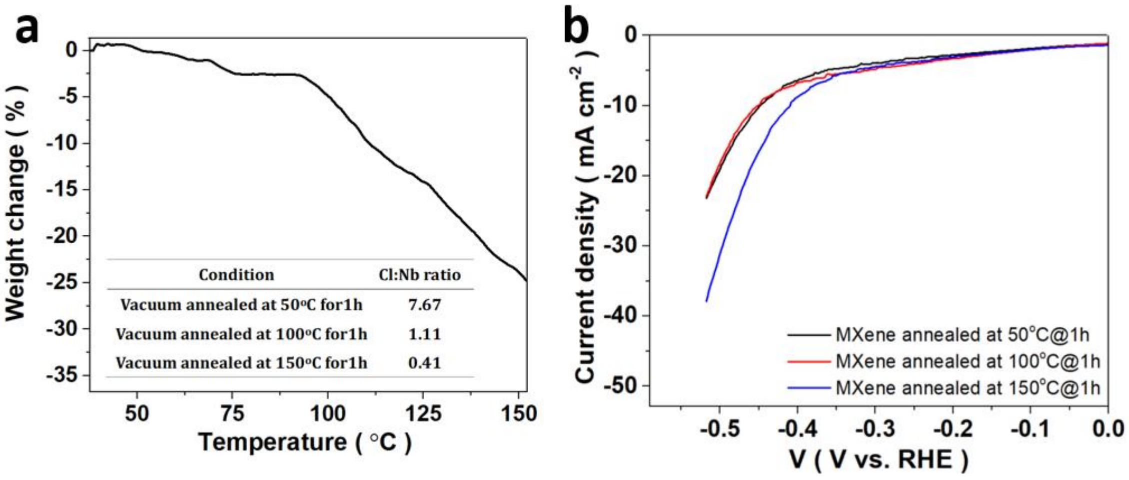

Figure S2. The effect of vacuum calcination on MXene. The thermogravimetric analysis and linear sweeping voltammetry curve of Nb_2CT_x MXene after vacuum annealing. The inset table is the chloride to niobium (Cl:Nb) ratio from the EDX result of Nb_2C samples.

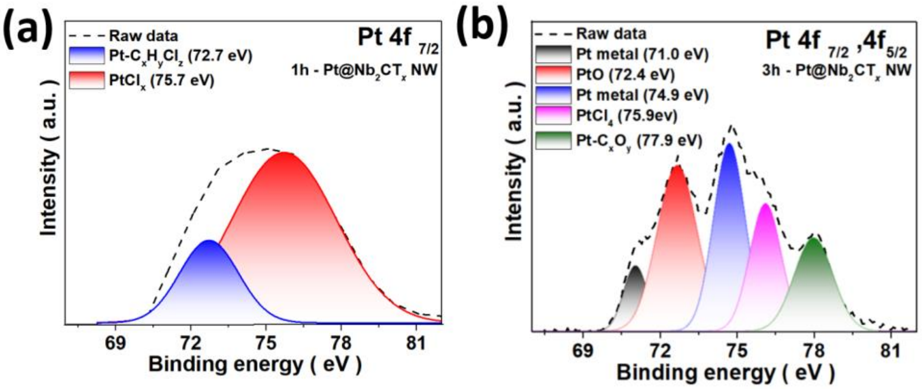

Figure S3. The XPS pattern of Pt in 4f orbitals. The Pt 4f regions of the *ex-situ* XPS spectra of (a) 1h-Pt@Nb_2CT_x NW and (b) 3h-Pt@Nb_2CT_x NW.

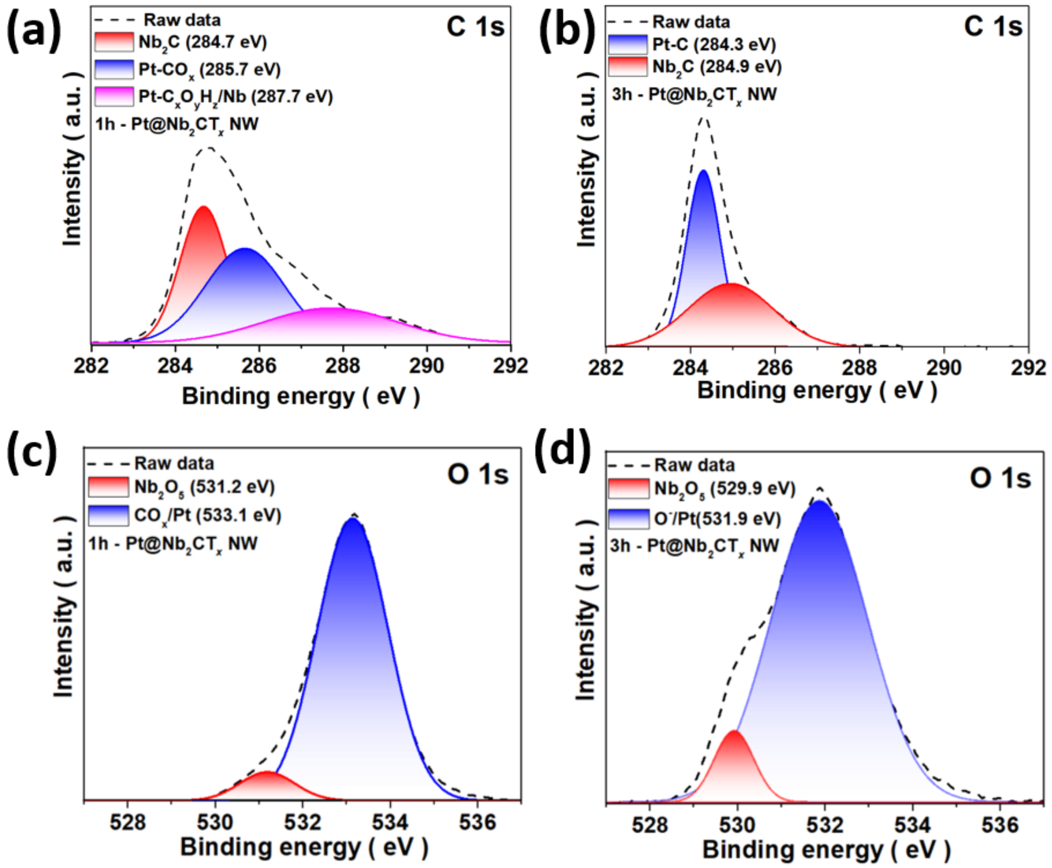

Figure S4. XPS result for Pt@Nb$_2$CT$_x$ NW. C 1s for Pt@Nb$_2$CT$_x$ NW at (a) 1h and (b) 3h. O 1s states XPS spectra of Pt@Nb$_2$CT$_x$ NW samples annealed at (c) 1h and (d) 3h.

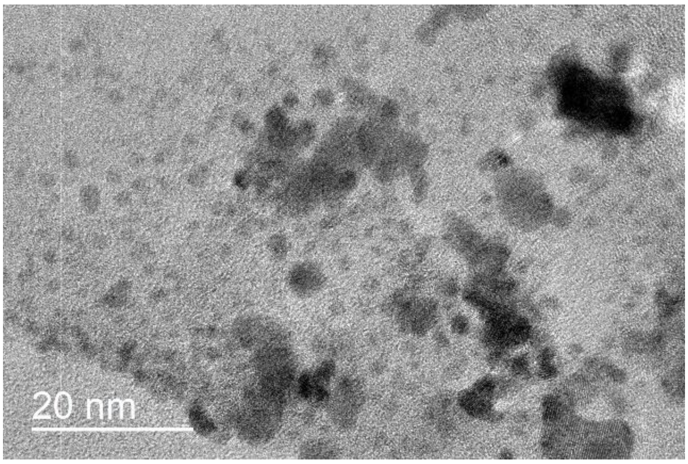

Figure S5. The TEM image of the 6h-Pt@Nb$_2$CT$_x$ sample catalyst.

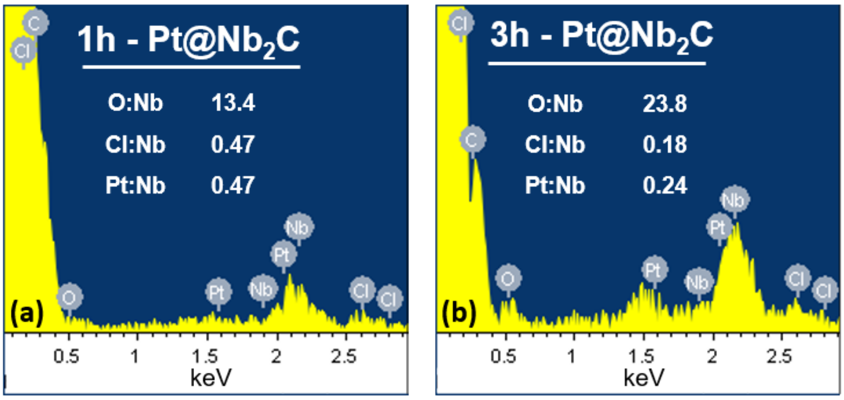

Figure S6. EDX analysis. Measurements obtained from (a) 1h-Pt@Nb_2CT_x and (b) 3h-Pt@Nb_2CT_x samples.

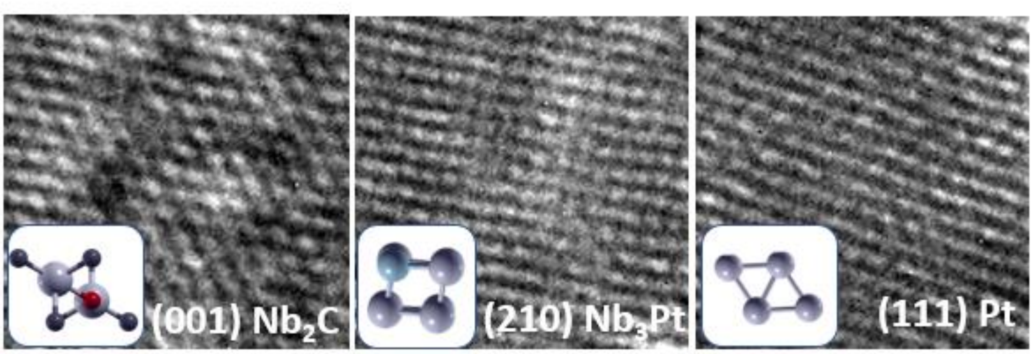

Figure S7. The HRTEM image of materials observed in 1h-Pt@ Nb_2CT_x

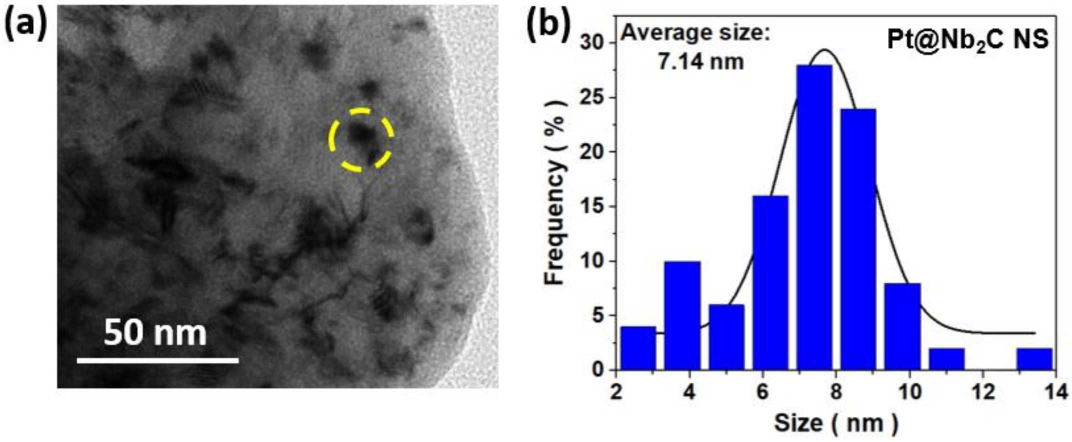

Figure S8. The morphological investigation on nanoparticles. (a)TEM image for Nb_2CT_x NS. (b) The corresponding statistical result of the size of the Pt nanoparticles on Nb_2CT_x NS.

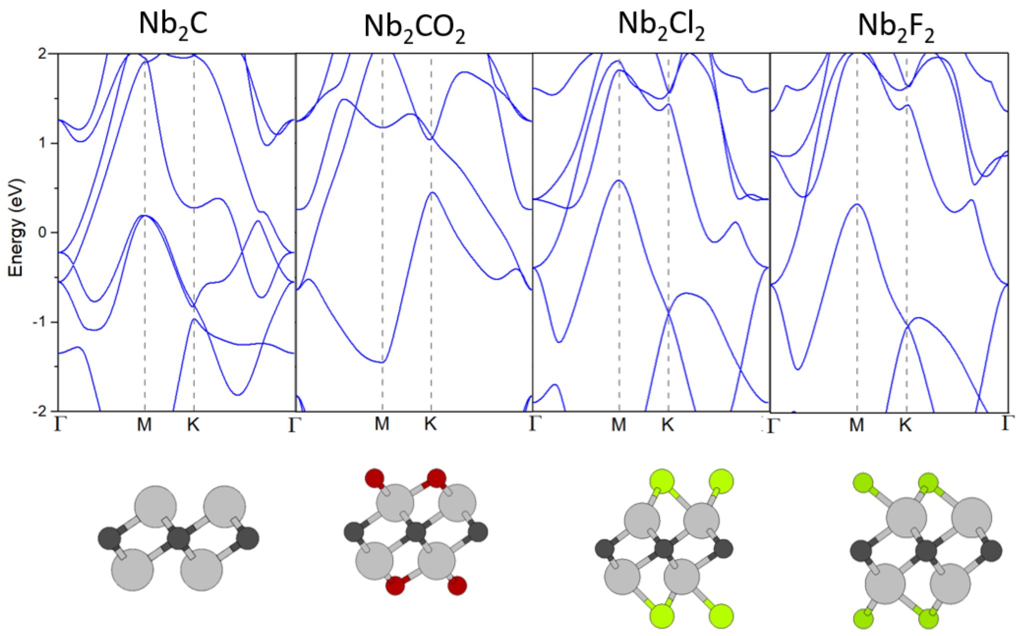

Figure S9. Proposed model for the Nb_2CT_x. Band structure of bare, oxygen-terminated, chlorine-terminated and fluorine-terminated MXene. The corresponding models of MXene are presented at the bottom of the band structures. Color code: Niobium, grey; oxygen, red; chlorine, light green; fluorine, deep green. The Fermi level is set to zero.

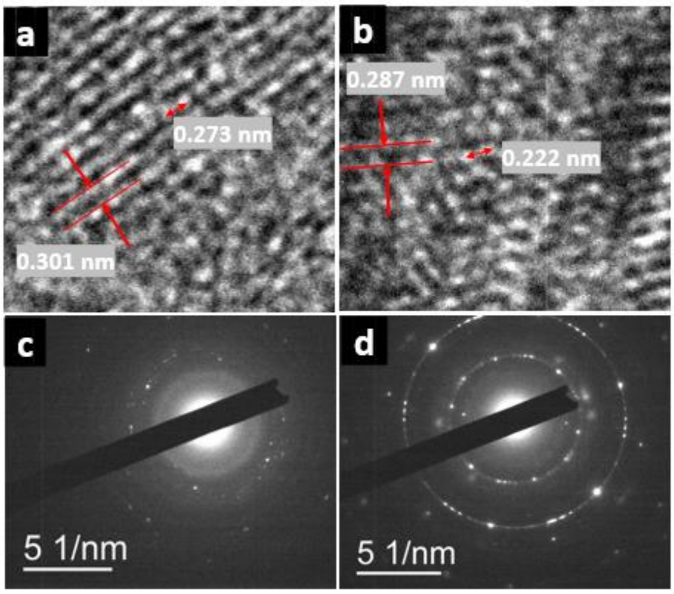

Figure S10. The in-plane lattice size difference between HF-free MXene and HF-etched MXene. (a) TEM images for HF-free MXene with larger in-plane *a* lattice parameter while (b) HF-etched MXene holds a smaller in-plane *a* lattice parameter. (c) and (d) are the SAED images for HF-free and HF-etched MXene, respectively.

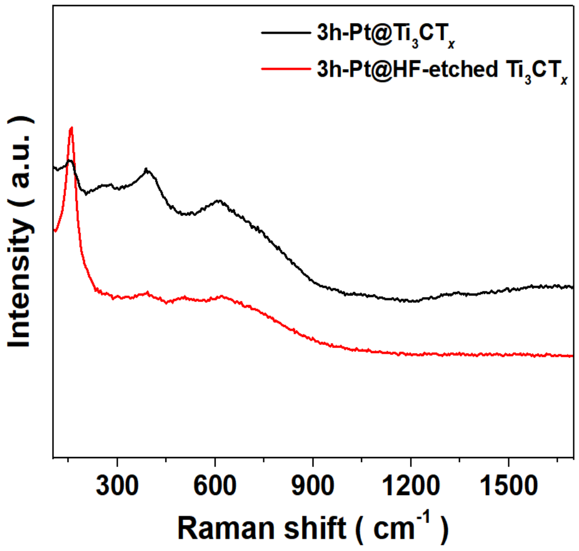

Figure S11. The Raman pattern of various catalysts. (a) Raman peaks of Pt NPs on 3h-Pt@HF-$Ti_3C_2T_x$ and 3h-Pt@$Ti_3C_2T_x$.

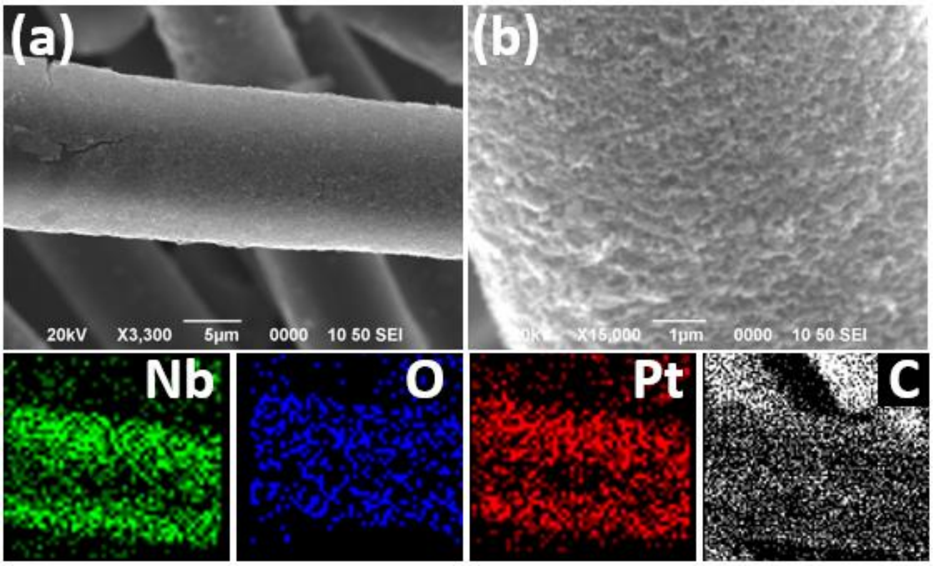

Figure S12. Morphology and elemental analysis for 3D-Pt@Nb_2CT_x NW. (a)Under low magnification and (b) high magnification.

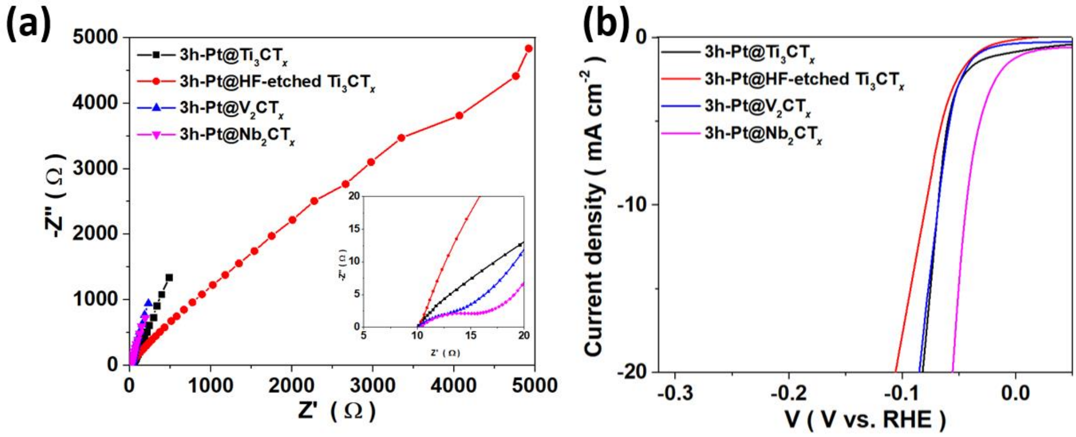

Figure S13. Electrochemical characterization and measurements for 3h-Pt@HF-Ti_3CT_x, 3h-Pt@Ti_3CT_x, 3h-Pt@V_2CT_x and 3h-Pt@Nb_2CT in acidic solution. (a) EIS curves and (b) LSVs.

Supporting references:

[1] N. Mahmood, Y. Yao, J.W. Zhang, L. Pan, X. Zhang, J.J. Zou, *Adv. Sci.* **2018**, 5, 1700464.

[2] S.-Y. Pang, W.-F. Io, L.-W. Wong, J. Zhao, J. Hao, *Adv. Sci.* **2020**, 7, 1903680.

[3] Z. Li, Z. Qi, S. Wang, T. Ma, L. Zhou, Z. Wu, X. Luan, F.-Y. Lin, M. Chen, J.T. Miller, *Nano Lett.* **2019**, 19, 5102-5108.

[4] Y. Yuan, H. Li, L. Wang, L. Zhang, D. Shi, Y. Hong, J. Sun, *ACS Sustainable Chem. Eng.* **2019**, 7, 4266-4273.

[5] J. Zhang, Y. Zhao, X. Guo, C. Chen, C.-L. Dong, R.-S. Liu, C.-P. Han, Y. Li, Y. Gogotsi, G. Wang, *Nat. Catal.* **2018**, 1, 985-992.

[6] H. Yin, S. Zhao, K. Zhao, A. Muqsit, H. Tang, L. Chang, H. Zhao, Y. Gao, Z. Tang, *Nat. Commun.* **2015**, 6, 1-8.
